# Prescribing Narcotics for Pain: Reconsider the Fifth Vital Sign

**DOI:** 10.1097/AS9.0000000000000186

**Published:** 2022-07-21

**Authors:** Ronald A. Charles, Soozan Abouhassan, Heather McFarland, Peter J. Pronovost

**Affiliations:** * Department of Surgery, Case Western Reserve University, School of Medicine, Cleveland, OH; † Division of Colon & Rectal Surgery, University Hospitals Cleveland Medical Center, Cleveland, OH; ‡ Department of Anesthesiology and Perioperative Medicine, University Hospitals, Cleveland Medical Center, Cleveland, OH; § Department of Anesthesia and Perioperative Medicine, University Hospitals, Cleveland, OH; ∥ Department of Anesthesiology and Critical Care Medicine, Case Western Reserve University, School of Medicine, Cleveland, OH.

Despite significant efforts from federal and state authorities, the opioid epidemic continues to devastate public health in the United States. On November 17, 2021, the Centers for Disease Control and Prevention National Center for Health Statistics released provisional data reporting >100,000 drug overdose deaths in the United States within the 12-month span ending April 2021,^1^ the highest number recorded in 1 year. The etiology of this epidemic is undeniably complicated and multifactorial. However, we must acknowledge that isolation, along with suffering seen from the COVID-19 pandemic has played a critical role in the exacerbation of this problem. Furthermore, we know that the lethality of drugs used has greatly increased because many drug cocktails are now laced with fentanyl, a potent and often deadly drug.

Despite the admitted complexity of the opioid epidemic, 1 causative factor that rarely gets acknowledged and discussed is the role we, as healthcare providers, play in this problem when we over prescribe narcotics to surgical patients. Seventy-one percent of people inappropriately using opioids have received the drugs through diversion of an appropriately obtained prescription, and in 55% of these cases, the drug is obtained from a family member or friend who has excess pills.^2^ In this article, we touch on the concept of pain as the fifth vital sign, examine some studies comparing opioid prescribing habits in the United States versus some European countries, and conclude by recommending a call to action for surgical programs around the country.

Those of us who were medical students in the 1990s and early 2000s in the United States will likely vividly remember being taught that pain was the “5th vital sign.” We were encouraged to treat pain with narcotic therapy to decrease any unnecessary suffering of our patients. In 1980, a short letter to the editor was published in *New England Journal of Medicine* titled “Addiction rare in patients treated with narcotics.”^3^ This thinking at the time equated pain control with opiate prescription, largely ignoring nonopiate drugs and nonpharmacologic therapies. These teachings have perhaps contributed to significant differences in narcotic prescribing practices in the United States.

Narcotic prescribing is dramatically higher in the United States than in some European countries. Lindenhovius et al^4^ retrospectively compared patients who underwent fracture repair in a level I trauma center in the United States versus the Netherlands to determine opioid prescribing practices. After discharge for hip repair, 77% of American and 0 Dutch patients received opioids, and for ankle repair, 82% of American and 6% of Dutch patients received opioids. Given the proclivity to prescribe narcotics after discharge from surgery, the overall proportion of adult patients with excess narcotics ranges from 67% to 92% with the proportion of unused pills ranging from 42% to 71%.^5^

Similarly, Ladha et al^6^ studied 223,834 opioid-naive patients undergoing 4 common surgical procedures in the United States, Canada, and Sweden; the results were quite profound. The authors found that for every procedure examined, the United States and Canada filled narcotic prescriptions at a sevenfold higher rate than Sweden. Furthermore, the mean quantity of narcotics dispensed was significantly higher in the United States.^6^ There is no evidence that patients in any of these studies suffered more pain.

These data tell a compelling story. Unwittingly and perhaps unknowingly, surgeons in the United States largely give too many patients narcotics, give too many pills in a prescription, and likely significantly underuse nonopiate pharmacologic therapies and nondrug therapies. Moreover, overprescribing is largely invisible as opiate prescribing is rarely routinely monitored as a quality metric to be used in quality improvement efforts.

## A CALL TO ACTION

We propose a call to action to address this crisis. There needs to be ongoing quality assessments at the hospital level by continuous analysis of narcotic utilization in patients being discharged in the postoperative setting. This can be done with routine measurement of the percent of hospitalized patients discharged home with narcotics and the percent not on maximal nonopiate therapy and done by measuring the quantity of narcotics prescribed in morphine milligram equivalents (****MME). The Centers for Medicare & Medicaid Services National Surgical Quality Improvement Program can work on definitions for these measures, ensure standardized measurement, and transparently report the results. Health systems should monitor these metrics and share data with their board and community.

Utilization metrics are just part of the solution. We also need to provide guidelines and education for providers and patients. Develop hospital standards and guidelines for the use of narcotics and then provide education and training to all clinicians with prescribing authority on good narcotic stewardship. These guidelines should encourage the use of non-narcotic analgesic options, such as acetaminophen and ibuprofen. Although these medications have been available for a long time, their use in the United States during the perioperative period has waned over the decades. Ample data has shown the efficacy of non-narcotic analgesics for pain control and a reduction in the quantity of opioid-based narcotics needed for pain management.^7,8^

Patients must also be educated in the perioperative period to prepare them and set appropriate expectations, as surgery and the resultant postoperative pain can be very distressing for many patients. Providing them with adequate knowledge and counseling for how the health care team will use a multimodal approach to manage their pain will likely lead to less stressful and anxiety-laden experience, and thus, lead to better compliance with narcotic consumption.^9^ Health systems should complete their call to action by implementing known programs that have been shown to reduce narcotic use by patients. Programs such as enhanced recovery pathways, which use an evidence-based approach to the care of surgical patients by implementing multiple preoperative, intraoperative, and postoperative interventions, have reduced narcotic consumption by patients.^10^ Finally, the Federal government and foundations should fund research on alternatives to narcotics. As we look to the future, we must begin to address how we can be part of the solution, this call to action provides a start.

## ACKNOWLEDGMENTS

The authors thank Christine G. Holzmueller, MS, for reviewing and editing the manuscript.
